# Supplementary material for: Sustained-input switches for transcription factors and microRNAs are central building blocks of eukaryotic gene circuits
Source: Genome Biol. 2013 Aug 23;14(8):R85. doi: 10.1186/gb-2013-14-8-r85 (PMC4054853; doi:10.1186/gb-2013-14-8-r85)
Supplement: Additional file 5 — HTML Browsable Motif Output. Zipped folder containing all WaRSwap and FANMOD motif output, viewable in a web browser. [file gb-2013-14-8-r85-S5.ZIP › HTML_browsable_motif_output/WaRSwap_all_tair9/sigs_WSWRSLDraw-2000_all.pvals.heatmaps.html/motif_id_38_000100110_tftype_all_upstream_-1000_0.html]

```
BG_MODEL = Rnew2
MOTIF_ID = 38_000100110
TF_TYPE = all
UPSTREAM = -1000_0


PVals
FN_0.2	FN_0.4	FN_0.6	FN_0.8
dg_60.genes	0.982	0.952	0	0.016
dg_70.genes	0.98	0.951	0	0.021
dg_80.genes	0.978	0.955	0	0.02

ZScores
FN_0.2	FN_0.4	FN_0.6	FN_0.8
dg_60.genes	-1.984	-1.719	4.544	2.101
dg_70.genes	-1.97	-1.673	4.666	2.035
dg_80.genes	-1.929	-1.691	4.618	2.118

StDevs
FN_0.2	FN_0.4	FN_0.6	FN_0.8
dg_60.genes	4959.082	1406.993	160.7	25.017
dg_70.genes	5025.885	1413.156	159.492	25.626
dg_80.genes	5023.226	1435.336	160.349	24.659
```
